# Job Characteristics and Serum Lipid Profile in Japanese Rural Workers: The Jichi Medical School Cohort Study

**DOI:** 10.2188/jea.13.63

**Published:** 2007-11-30

**Authors:** Akizumi Tsutsumi, Kazunori Kayaba, Shizukiyo Ishikawa, Tadao Gotoh, Naoki Nago, Seishi Yamada, Masafumi Mizooka, Kenichiro Sakai, Shinya Hayasaka, the Jichi Medical School Cohort Study Group

**Affiliations:** 1 Okayama University School of Medicine and Dentistry, Social and Environmental Life Sciences, Social Medicine and Longevity Sciences, Hygiene and Preventive Medicine.; 2 Department of Community and Family Medicine, Jichi Medical School.; 3 Department of Internal Medicine, Wara National Health Insurance Hospital.; 4 Tsukude National Health Insurance Clinic.; 5 Hiroshima Prefectural Setoda Hospital.; 6 Akaike Town Hospital.

**Keywords:** psychological stress, work, lipid, Japanese, gender

## Abstract

To observe the association between adverse psychosocial job characteristics, measured by the Karasek job demand-control questionnaire, and a lipid profile, cross-sectional analyses were performed for a Japanese rural working population. The study population comprised 3,333 male and 3,596 female actively employed workers, aged 65 years and under. Among men, higher psychological demands were associated with high total cholesterol levels, with an adjusted difference from the top to bottom tertiles of 3.3 mg/dl (*F* = 3.03; *p* = 0.048). High demands were also positively associated with the total/HDL cholesterol ratio (*F* = 3.94; *p* = 0.020). Neither job control nor job strain (the ratio of demands to control) was associated with any of the lipid levels in either gender. A psychologically demanding job may be associated with an unfavorable lipid profile, but the impact of job strain on atherogenic lipids is negligible.

Evidence has been accumulating that adverse psychosocial job characteristics may predict the onset of cardiovascular diseases. In occupational stress research, the job demand-control model is currently most prevalent among those that demonstrate such evidence.^1^^-^^3^ The hypothesis of the job demand-control model proposes that employees with a combination of high psychological job demand and low control over their job (i.e., job strain) are at risk of developing illnesses.^4^ Although the combined effect of the demand and control components is of central interest to this model, its individual components may also predict a worker’s stress-related health outcome.

Despite the numerous empirical studies that show an association between job strain or components and cardiovascular diseases, the physiologically mediating mechanisms remain unresolved. Metabolic disorders, such as an unfavorable lipid profile, might be one of the mediators,^5^^,^^6^ but the evidence on the association between job strain and atherogenic lipids is far from definite. Theorell et al.^7^ showed that there was a significant correlation between the ratio of job demands to influence over the job and total cholesterol level. Niedhammer et al.^8^ showed a high prevalence of reported hyperlipidemia in men exposed to both high job demands and low support, and in women with low decision latitude (low job control). This finding about women was replicated in other studies.^9^^,^^10^ However, there are many other studies in which no association between job strain or its components and atherogenic lipids was demonstrated.^11^^-^^17^

Outside of Europe and the United States, very few studies have been conducted to investigate this association.^2^^,^^13^^,^^14^ Furthermore, most of the earlier studies were conducted on workers employed in big enterprises or in urban settings. To the best of our knowledge, except for some epidemiologic studies of which study population was national representative sample and presumably included pre-industrial workers,^16^ studies that investigated occupational stress involving large populations of workers in farming, forestry and fisheries are scarce. Even in such a study, attempts have not been made to explore the association between job characteristics and atherogenic lipids specifically for pre-industrial workers. Accordingly, the aim here was to observe the association in Japanese workers from a large community-dwelling sample in non-urban areas.

## METHODS

The aim of the Jichi Medical School Cohort Study was to investigate the risk factors of cardiovascular disease in Japan. Data were collected between 1992 and 1995. Ultimately, 12,490 Japanese (4,911 men and 7,579 women) from 12 rural communities located across Japan participated.^18^ In accordance with the provisions of the Health and Medical Service Law for the Aged, a mass screening examination program concerned with the risk factors for cardio-cerebrovascular disease have been conducted since 1983: The law requires municipal governments to manage the programs efficiently and offer them to all residents who are willing to participate. The target subjects vary according to each community, from all the residents to those who are not offered physical examinations at their workplaces or elsewhere including the subjects of the National Health Insurance. Residents aged 40-69 years were the subjects for the mass screening examination program in 8 of the 12 communities, those aged 30 years and older in one, and adults for other age groups were included in the rest. Accordingly, the cohort for the study was to include residents between 40 and 69 years of age living in the first 8 communities and between 30 and 69 years in the other 4 initially. In each community, the local government office sent letters to all potential participants inviting them to take part in the program. The invitation mentioned that persons who were visiting hospitals or clinics because of cardiovascular disease did not have to take the examination. People other than those in the above-defined age groups (*n* = 282 for the younger age group and 696 for those over 69) as well as those who voluntarily applied for the program participated in the study and are included in the database. The overall response rate was 65.4%, whereas the response rate based on the intended subjects between 40 and 69 years was 62.7%.

In this analysis, the study population was limited to actively working men and women who were not older than 65 because the aim of this analysis was to observe the association between job characteristics and serum lipid profiles. A total of 6,929 participants (3,333 men and 3,596 women) who were employed and had filled out the questionnaire were subjected to the present study. The following occupations were included: farming and forestry (*n* = 1,074 men, 1,253 women), fisheries (248, 37), security (21, 1), transportation (94, 4), construction (629, 86), production (345, 680), business (258, 378), office work (200, 352), professional (201, 207), and the service industry (263, 598). The first six were designated blue-collar jobs and the next four, white-collar. With regard to employment status, workers who classified themselves as administrators or self-employed were labeled managerial. More than 99% of the workers were either self-employed or employed by companies with less than 300 employees, which may reflect the current industrial structure in Japan.^19^ However, it should be noted that the under-representativeness for younger working population precludes the generalizability of the results. Some who were employed part-time may have been included in the study population but this was not ascertained.

### Procedure

Socio-demographic and behavioral variables were obtained by using a standardized questionnaire, which included information on occupational environment, age, marital status, educational attainment, dietary habits, smoking habits, alcohol consumption, physical activity, and medical history. The questionnaire was distributed to the subjects beforehand to complete on their own. Informed consent was obtained from all prospective participants.

Job characteristics were derived by using a Japanese version of the demand-control questionnaire^20^ originally developed by Theorell et al.^21^ The psychometric property of the questionnaire has been reported elsewhere.^22^ Those job characteristics, job control and psychological demands, were defined on two scales. The job control was defined as the sum of two subscales that were given equal weight: (1) skill discretion, measured by four parameters (possibility for learning new things, skills required by the job, requirement for creativity, and repetitious nature of the work); and (2) autonomy for decision making, measured by two parameters (right to make one’s own decisions and freedom in choosing the manner by which the work is performed). The second scale, psychological job demands, was defined by five parameters (speed in completing work, degree of difficulty of the work, excessive workload, insufficient time allowed to complete work, and conflicting demands). All questions were scored on a Likert scale of 1 to 4. Cronbach’s coefficient alpha for the job control index and for the psychological demands index was 0.64 and 0.70, respectively. Job strain was defined as the ratio of demands to job control. The participants were grouped into one of three strata for each job characteristic index (low, medium or high) based on tertiles, defined according to the distribution of scores in the total working population, then separately for men and women.

The following socio-demographic and behavioral variables were used as possible covariates. The participants were grouped by age into four categories: under 39 years, 40-49, 50-59, and 60-65. Marital status was coded as currently married or unmarried. Educational attainment was categorized into two strata: lower or higher than the level of compulsory education.

The dietary habits of the participants were ascertained by employing a food frequency questionnaire, which was composed of 30 different foods most likely to be consumed, each on a graded five-point Likert scale.^23^ The items were subjected to a factor analysis with Varimax rotation; and with regard to the dietary pattern, three additive scales that were composed of factors with eigenvalues greater than 1.5 were created. An item was included in a scale if it had a loading of 0.40 or above on one factor but less than 0.39 on any other factor. These cutoff points were selected to ensure that no item was included on more than one scale. They were: vegetable type (food preference for green vegetable, yellow-green vegetables, potatoes, fruits, tofu, seaweed, oranges, beans, and dried fish); meat type (ham, pork, beef, chicken, and fish-paste); and Western type food (bread, butter, rice (reversed score), salty food (reversed score), miso soup (reversed score), and yogurt). The alpha coefficients for the three scales ranged from 0.55 to 0.76. A participant was placed in one of three linear strata for the food frequency pattern based on the tertile in which his/her score fell. Analysis of covariance, adjusted for possible confounders, revealed that frequent consumption of vegetables was associated with a lower blood pressure (systolic and diastolic, *F* = 11.4, p< 0.001, and *F* = 6.8, *p* = 0.001, respectively), while frequent consumption of Western foods was associated with a higher diastolic blood pressure (*F* = 4.9, p = 0.008). There were also positive associations between frequent consumption of Western foods and total cholesterol (*F* = 18.0, p < 0.001) and between frequent consumption of vegetables and HDL cholesterol levels (*F* = 3.6, *p* = 0.028). The group with the lowest frequency of meat consumption exhibited the lowest total cholesterol levels (*F* = 7.5, *p* = 0.001) but the pattern was nonlinear (M. Yoshimura, personal communication).

Smoking habits were classified as “lifetime non-smoker”, “exsmoker”, “1-20 cigarettes per day”, or “21+ cigarettes per day” for men; and “lifetime non-smoker”, “ex-smoker”, or “current smoker” for women. The total average amount of alcohol consumed was calculated in grams per day, after taking into account the frequency, amount, and alcohol content for specific beverages. Alcohol consumption was categorized into “non-drinker”, “<1 *go* daily” (*go*; a traditional Japanese alcohol unit, 1 *go* = 28.9 grams of alcohol), “1-3 *go* daily” (28.9-86.6 g), or “3+ *go* daily” (≧86.7 g) for men; and “non-drinker”, “<1 *go* daily”, or “1+ *go* daily” for women. The physical activity index, which was developed in the Framingham Study,^24^ was calculated by totaling the hours at each level of activity and multiplying this by a weight that was based on the oxygen consumption required for that activity. We computed the physical activity indices based on reported activity during an ordinary day taking into account physical activity at work. The index was categorized into three strata: low (<29), medium (29-36), and high (37+).

Women who reported that they were in a postmenopausal stage were defined as such, whether it was natural or surgically induced. Although contraceptive use and postmenopausal hormone replacement therapy are possible modifiers of serum lipid profiles,^25^^,^^26^ they were not taken into consideration here because of their low prevalence among Japanese.^27^^,^^28^ Other than those variables, seasonal variations can occur in cholesterol levels.^29^ Only 8% (*n* = 409) of the blood sample was drawn during the winter season (November through February): the effect of adjusting for this was negligible. The prevalence of hypertension in those under antihypertensive therapy was low (7% for men, 8% for women); and so was that for those under medication for hyperlipidemia (1% for both men and women). Adjusting for those under medication (possible lipid-altering medications) did not alter the results; thus these variables were not considered in the analyses.

The physical examinations took place in each community. Height was measured without shoes;body weight was recorded with the subject clothed; and 0.5 kg in summer or 1 kg in the other seasons was subtracted from the recorded weight. The body mass index (BMI) was calculated as weight (kg)/height (m)^2^. BMI readings were categorized into tertiles, based on the total sample distribution (<21.6 kg/m^2^, 21.6-23.9 kg/m^2^, or 24.0+ kg/m^2^).

While the subject was seated, blood samples were drawn from the antecubital vein with the minimal use of a tourniquet. Specimens were collected in siliconized vacuum glass tubes containing no additives. The tubes were centrifuged at 3,000 G for 15 minutes at room temperature. Total cholesterol was measured by an enzymatic method (Wako, Osaka, Japan). High-density lipoprotein (HDL) cholesterol was measured by the phosphotungstate precipitation method (Wako). Lipoprotein (a) levels were measured by using an enzyme-linked immunosorbent assay kit (Biopool, Uppsala, Sweden). Blood variables were measured at the Central Laboratory of SRL (Tokyo, Japan), a commercial hematology laboratory, where the measurements were all standardized by the Lipid Standardization Program, Center for Disease Control and Prevention, Atlanta, Georgia. All the lipid profiles were determined except in a single community (*n* = 450), where lipoprotein (a) was not measured.

As another outcome, a ratio of serum total cholesterol divided by HDL cholesterol was computed. According to Criqui and Golomb,^30^ this ratio provides a more precise estimate of coronary risk than each variable considered individually; and this ratio has been used in several studies as a coronary risk indicator for both men and women.^25^^,^^31^

### Statistics

All analyses were performed separately for men and women. Means, standard deviations, and 95% confidence intervals are given for the serum lipid profile data. Then a series of cross-tabulations of job-characteristics and socio-demographic/behavioral variables was performed. The chi-square test was conducted and a linear trend for the effect of job characteristics was assessed by computing the *p* value for trend. Associations between job characteristics and serum lipid levels were assessed by adjusting for possible covariates, and the adjusted means for three job characteristic levels are shown. In the statistical tests, the distributions for the lipoprotein (a) and the total/HDL cholesterol ratio levels were skewed. Therefore natural logarithms were used for these variables. For comparison, however, arithmetic means are displayed. The multivariate analyses were repeated on stratified subgroups; the pre-industrial occupations (farming, forestry and fisheries) and the other post-industrial occupations, to see if the patterns and findings observed for the full study population were the same across the occupations of interest.

Additional multivariate tests were done by using cutoff points, which were established in a guideline for Japanese that indicated the thresholds of increased risk for cardiovascular diseases.^32^

Logistic regression analyses were conducted using the following criteria as dependent variables: total cholesterol 220+ mg/dl, HDL cholesterol < 40 mg/dl, and lipoprotein (a) 40+ mg/dl. For the total/HDL cholesterol ratio, the upper tertile was chosen as a cutoff point related to the distribution of the data.^33^ Categorical variables including the job characteristics were represented by dummy indicators in the analyses.

All tests were two-tailed and a value where *p* < 0.05 was considered statistically significant. SPSS^®^ for Windows, release 6.1, was used for the statistical analyses.

## RESULTS

The serum lipid levels of the study population are displayed in Table 1.

**Table 1.** Lipid profiles of active workers aged 65 and under, JMS Cohort Study, 1992-1995.

	n	Mean	SD	95% CI
Men				
Total cholesterol (mg/dl)	3293	185.6	34.3	(184.4, 186.8)
HDL cholesterol (mg/dl)	3294	48.8	13.3	(48.4, 49.3)
Lipoprotein (a) (mg/dl)	2834	18.5	17.6	(17.9, 19.2)
Total/HDL cholesterol ratio	3293	4.06	1.33	(4.02, 4.11)

Women				
Total cholesterol (mg/dl)	3560	192.6	34.3	(191.5, 193.7)
HDL cholesterol (mg/dl)	3560	53.0	12.5	(52.6, 53.4)
Lipoprotein (a) (mg/dl)	3204	21.1	20.0	(20.4, 21.8)
Total/HDL cholesterol ratio	3560	3.82	1.11	(3.79, 3.86)

SD: standard deviation

CI: confidence interval.

Table 2 shows the sex-specific population profiles according to job characteristics. In both sexes, psychological demands were higher in the younger age groups and managers than in the respective others. High demands were associated with higher levels of alcohol consumption and physical activity. Men with high demands were married and frequent meat consumers. As for women, psychological demands were more prevalent in the blue-collar and the pre-menopausal women than in the respective counterparts. Job control was lower in the less educated, blue-collar, and subordinates, and low control was associated with low levels of vegetable consumption, physical activity, and BMI. Men with high job control were younger, married, and had high consumption of cigarettes and western foods, while women with high control had meat frequently. Both male and female blue-collar workers were exposed to job strain more frequently than white-collar workers. Men exposed to job strain were less educated and subordinate employees, and with high levels of meat consumption and physical activity. Women with job strain were younger and had vegetables less frequently.

**Table 2.** Socio-demographic and behavioral profiles according to job characteristics, active workers aged 65 and under, IMS Cohort Study, 1992-1995.

		Psychological demands							Job control							Job strain						
		
		Low		Middle		High		p	High		Middle		Low		p	Low		Middle		High		p
								
		n	%	n	%	n	%		n	%	n	%	n	%		n	%	n	%	n	%	
Men																						
Age	<40	111	12	145	15	189	15	<0.001	145	15	154	15	148	12	<0.001	131	13	156	15	156	14	0.106
	40-49	226	24	282	29	420	33		362	37	292	28	287	24		278	28	321	31	324	29	
	50-59	277	29	296	31	415	33		291	30	342	33	359	30		274	28	328	31	373	33	
	60-65	345	36	248	26	250	20		184	19	247	24	411	34		304	31	245	23	280	25	
Marital status	Married	854	89	878	91	1174	93	0.007	916	94	949	92	1058	88	<0.001	898	91	958	92	1020	90	0.535
	Unmarried	102	11	92	10	94	7		63	6	82	8	142	12		87	9	89	9	108	10	
Educational attainment	Higher than compulsory	563	59	565	59	791	63	0.065	654	67	607	59	673	56	<0.001	618	63	645	62	641	57	0.006
	Less than compulsory	391	41	401	42	471	37		322	33	420	41	525	44		363	37	402	38	480	43	
Type of job	White-collar	268	28	258	27	382	30	0.242	319	33	303	29	288	24	<0.001	309	31	293	28	299	26	0.013
	Blue-collar	691	72	713	73	892	70		663	68	732	71	917	76		678	69	757	72	834	74	
Employment status	Managerial status	396	46	475	55	735	63	<0.001	596	70	555	60	465	41	<0.001	502	59	526	55	562	53	0.026
	Subordinates	460	54	396	46	430	37		257	30	378	41	659	59		355	41	430	45	490	47	
Vegetable diet pattern	Low	316	35	311	34	386	33	0.767	277	30	331	34	409	37	0.005	327	35	310	31	367	35	0.209
	Middle	282	32	320	35	426	36		318	35	348	36	364	33		282	31	359	36	376	36	
	High	296	33	276	30	370	31		322	35	283	29	348	31		314	34	317	32	306	29	
Meat diet pattern	Low	319	34	298	32	371	30	0.045	296	31	313	31	390	33	0.753	330	34	307	30	341	31	0.028
	Middle	324	35	339	36	448	36		344	36	380	38	396	34		353	37	350	34	400	36	
	High	292	31	310	33	422	34		309	33	319	32	393	33		280	29	359	35	373	34	
Western diet pattern	Low	285	32	283	32	371	31	0.697	266	30	290	30	382	34	0.010	279	31	290	30	357	34	0.158
	Middle	289	33	312	35	387	33		292	33	327	34	372	33		311	34	325	34	346	33	
	High	315	35	296	33	425	36		333	37	346	36	363	33		323	35	348	36	355	34	
Smoking habits	Never smoker	215	23	204	21	279	22	0.255	202	21	234	23	265	22	0.029	209	21	224	21	254	23	0.237
	Ex-smoker	221	23	247	26	310	24		236	24	248	24	301	25		231	24	248	24	294	26	
	Current, 1-20/day	364	38	366	38	414	33		339	35	349	34	453	38		369	38	379	36	381	34	
	Current, 21+/day	155	16	149	15	268	21		204	21	196	19	181	15		175	18	194	19	200	18	
Alcohol consumption	Never and ex-drinker	225	25	195	21	240	19		184	19	216	21	266	23	0.179	217	23	206	20	231	21	0.158
	Current, <29 g/day	259	28	277	29	379	30		303	32	304	30	315	27		282	30	310	30	314	29	
	Current, 29-87 g/day	355	39	387	41	500	40		372	39	395	39	481	42		368	39	417	41	444	40	
	Current, 87+ g/day	79	9	82	9	129	10		100	10	96	10	95	8		85	9	91	9	111	10	
Physical Activity Index	<29	185	19	170	18	241	19	<0.001	164	17	195	19	243	20	0.007	183	19	191	18	216	19	0.005
	29-36	434	46	341	35	417	33		369	38	353	35	471	39		422	43	399	38	358	32	
	37+	335	35	452	47	601	48		440	45	476	47	481	40		377	38	456	44	540	49	
BMI (kg/m^2^)	<21.6	303	32	288	31	380	30	0.328	257	27	300	30	419	36	<0.001	287	30	305	30	371	33	0.354
	21.6-23.9	326	35	333	35	442	35		355	37	376	37	378	32		359	37	357	35	373	33	
	24.0+	305	33	323	34	427	34		348	36	336	33	377	32		318	33	353	35	372	33	

Women																						
Age	<40	103	10	145	13	117	9	<0.001	126	11	133	11	111	11	0.801	113	10	137	13	112	10	0.001
	40-49	295	29	346	32	438	34		379	32	387	32	320	31		303	28	378	35	378	32	
	50-59	340	33	390	36	517	41		464	39	406	34	388	38		373	34	376	35	482	41	
	60-65	292	28	206	19	204	16		225	19	282	23	203	20		295	27	194	18	200	17	
Marital status	Married	959	94	1009	93	1199	94	0.567	1111	93	1138	95	943	93	0.491	1008	94	1013	94	1097	94	0.999
	Unmarried	64	6	75	7	73	6		78	7	64	5	75	7		68	6	69	6	74	6	
Educational attainment	Higher than compulsory	559	55	646	60	720	57	0.378	729	61	677	56	537	53	<0.001	619	57	655	61	626	54	0.069
	Less than compulsory	465	45	438	40	550	43		461	39	528	44	475	47		461	43	425	39	541	46	
Type of job	White-collar	471	46	488	45	511	40	0.005	580	49	523	43	383	38	<0.001	522	48	509	47	418	36	<0.001
	Blue-collar	559	54	599	55	765	60		614	51	685	57	639	63		562	52	576	53	754	64	
Employment status	Managerial status	249	27	305	32	397	36	<0.001	400	40	309	29	251	26	<0.001	300	33	330	35	312	29	0.058
	Subordinates	662	73	646	68	720	65		591	60	751	71	705	74		612	67	621	65	760	71	
Vegetable diet pattern	Low	300	31	331	33	401	34	0.167	306	28	374	34	367	38	<0.001	278	28	345	34	391	36	<0.001
	Middle	364	38	360	36	426	36		399	36	400	36	356	37		378	37	362	36	396	37	
	High	303	31	316	31	349	30		399	36	344	31	235	25		354	35	307	30	297	27	
Meat diet pattern	Low	348	35	345	33	412	33	0.073	358	31	399	34	353	35	0.007	354	34	341	32	388	34	0.878
	Middle	372	37	381	36	425	34		423	36	411	35	367	37		374	35	394	37	397	35	
	High	286	28	337	32	413	33		385	33	367	31	283	28		328	31	327	31	366	32	
Western diet pattern	Low	357	38	369	38	447	38	0.127	397	37	414	38	371	40	0.333	361	37	367	37	421	39	0.683
	Middle	323	35	303	31	366	31		367	34	353	32	276	30		354	36	306	31	317	30	
	High	249	27	313	32	355	30		324	30	327	30	276	30		263	27	316	32	331	31	
Smoking habits	Never smoker	917	90	994	93	1136	91	0.782	1065	91	1097	92	909	91	0.935	969	91	970	91	1060	92	0.317
	Ex-smoker	31	3	19	2	29	2		23	2	34	3	26	3		26	2	27	3	25	2	
	Current smokers	68	7	58	5	83	7		82	7	60	5	69	7		71	7	70	7	66	6	
Alcohol consumption	Never and ex-drinker	725	73	735	70	819	67	0.009	776	68	831	72	696	71	0.158	751	72	707	68	782	69	0.175
	Current, <29 g/day	217	22	262	25	335	27		300	26	280	24	237	24		239	23	277	27	287	25	
	Current, 29+ g/day	51	5	51	5	68	6		67	6	51	4	52	5		50	5	58	6	60	5	
Physical Activity Index	<29	271	27	260	24	323	26	0.002	267	23	304	26	301	30	<0.001	267	25	270	25	312	27	0.114
	29-36	551	54	545	51	590	47		567	48	594	50	531	53		543	51	527	49	587	51	
	37+	190	19	267	25	343	27		350	30	287	24	169	17		260	24	273	26	251	22	
Menopausal status	Premenopausal	388	38	492	46	556	44	0.006	493	42	529	44	430	43	0.667	411	38	517	48	491	42	0.068
	Postmenopausal	632	62	580	54	705	56		688	58	663	56	581	58		661	62	554	52	668	58	
BMI (kg/m^2^)	<21.6	322	32	363	34	419	34	0.754	381	33	383	32	356	36	0.029	325	31	361	34	403	35	0.061
	21.6-23.9	347	35	366	35	408	33		372	32	400	34	349	35		364	34	364	34	374	33	
	24.0+	337	34	333	31	421	34		406	35	401	34	299	30		368	35	333	32	374	33	

Chi-square test was conducted for linear trends.

The numbers do not add up to the total number of subjects due to missing values.

The analysis of covariance showed significant differences across demand levels for total cholesterol and the total/HDL cholesterol ratio in men. Higher psychological demands were associated with a higher total cholesterol level, with an adjusted difference from the top to bottom tertile of 3.3 mg/dl (*F* = 3.03, *p* = 0.048). Higher demands were also associated with a higher total/HDL cholesterol ratio (*F* = 3.94, *p* = 0.020). The linear associations were confirmed by multiple regression analyses. However, neither job control nor job strain was associated with any lipid levels. No significant associations were found in women (Table 3).

**Table 3.** Associations between job characteristics and serum lipid profiles, active workers aged 65 and under, JMS Cohort Study, 1992-1995.

	Psychological demands				Job control				Job strain			
		
	Low	Middle	High	p	High	Middle	Low	p	Low	Middle	High	p
Men												
n	959	971	1274		982	1035	1205		987	1050	1133	
Total cholesterol (mg/dl)	185.4	184.9	188.7	0.048	188.6	186.7	185.0	0.140	185.9	188.0	185.9	0.388
HDL cholesterol (mg/dl)	48.6	48.6	48.0	0.451	48.2	47.9	48.7	0.402	48.2	48.2	48.5	0.780
Lipoprotein (a) (mg/dl)	19.6	17.3	18.3	0.148	17.1	19.3	18.8	0.283	18.3	17.9	18.9	0.443
Total/HDL cholesterol ratio	4.07	4.06	4.20	0.020	4.17	4.17	4.06	0.102	4.11	4.19	4.09	0.300

Women												
n	1030	1087	1276		1194	1208	1022		1084	1085	1172	
Total cholesterol (mg/dl)	192.7	192.9	193.3	0.934	193.6	191.7	194.2	0.275	192.8	192.5	193.6	0.789
HDL cholesterol (mg/dl)	52.6	52.8	52.6	0.949	52.5	52.7	52.8	0.886	52.5	52.2	53.1	0.363
Lipoprotein (a) (mg/dl)	20.0	21.1	21.0	0.106	20.3	21.5	20.6	0.421	20.9	20.2	21.2	0.381
Total/HDL cholesterol ratio	3.86	3.85	3.87	0.963	3.88	3.83	3.89	0.475	3.87	3.87	3.86	0.888

Due to selective missing values, the total number of subjects included in multivariate analysis was somewhat lower.

Analysis of covariance was performed adjusting for age, marital status, educational attainment, type of job, employment status, smoking hal alcohol intake, dietary patterns (vegetable pattern, meat pattern, and western pattern), physical activity index, and BMI.

In women, menopausal status was also adjusted for.

Observed findings of job demands and the both levels of total cholesterol and total/HDL cholesterol ratio in men were stronger in the post-industrial occupations (*F* = 2.90, *p* = 0.055 for total cholesterol, and *F* = 3.71, *p* = 0.025 for total/HDL cholesterol ratio, respectively). However, unexpected negative findings were also observed; male post-industrial workers with the lowest demands had the highest level of lipoprotein (a) (*F* = 3.38, *p* = 0.034) and female pre-industrial workers exposed to high strain had the highest level of HDL cholesterol (*F* = 5.20, *p* = 0.006).

Neither in the full study population nor in the stratified groups, did the logistic regression analyses reveal any significantly elevated risk of adverse job characteristics in unfavorable lipid categories (data not shown).

## DISCUSSION

In a Japanese male working population, a statistically significant difference of the total cholesterol levels was observed depending on the psychological job demands. High demands also had a significant association with the total/HDL cholesterol ratio. The well-known effects of dietary habits, smoking, alcohol drinking, physical activity, and relative weight on atherogenic lipids^25^^,^^34^^-^^37^ were taken into account in the statistical model. However, because the sample analyzed here appeared to have favorable lipid profiles,^30^^,^^38^ the magnitude of the observed effect, up to a 3% average difference of the total cholesterol levels, is not biologically meaningful. Besides, neither job control nor a combination of high demands and low control, represented by their ratio in this study, had expected associations with any lipid profile. Stratified analyses provided inconsistent results. These findings replicated most of those that appeared in previous studies,^11^^-^^17^ which show no significant associations between job strain or its major components and atherogenic lipids.

Our study was unique in that the study population was recruited from rural residents and included large numbers of pre-industrial workers. However, because of the sampling method, relatively older workers represented the study population. It was possible that the older workers, whose prevalence for hyperlipidemia were presumably high, accounted for the relation between psychological demands and high total cholesterol. In fact, the magnitude of the association between psychological demands and total cholesterol in men was stronger for the older workers (*F* = 3.46, *p* = 0.032 for workers aged 50-65 years, and *F* = 2.56, *p* = 0.078 but the pattern was nonlinear for workers aged 49 years and under, respectively). However, except for lipoprotein (a), mean lipid levels were generally better in the older men than in the others (the opposite was the case with female workers; data were not shown). Thus, at least in this male working population, the effect of age distribution for the lipid profiles on the results is not considered so large. However, to fill the gap of the knowledge, further studies are necessary including younger workers who appeared to be exposed to severer job characteristics (except for job control) in this study.

Failure to examine some possible biological variability in an individual’s cholesterol response could be a reason for the lack of significant associations in this study. First, individuals with cardiovascular problems were under-represented.^38^ This means that the association, if any, between adverse job characteristics and unfavorable lipid profiles may have been underestimated Cholesterol levels seem highly labile in some individuals^5^ and those with a high coronary risk may be susceptible to stress-induced alterations in their lipid metabolism.^6^^,^^39^ Second, lipid elevation appears to be more significantly affected by perceived stress than an objective stressful situation,^40^ whereas the participants were questioned about their job characteristics, not about their feelings about stress.^41^ Another possible explanation for the null association is relatively lower job demands level of our self-employed sample in the pre-industrial occupations.^22^^,^^42^ The job demands and job control for such an occupational group has been reported to be greater when compared to other occupations.^4^ It should be noted that the job control dimension had an unexpectedly low Chronbach’s alpha of 0.64, which may also have affected the results negatively. Lastly, because research has suggested that health behavior is in the causal pathway between job strain and cardiovascular disease and/or cardiovascular disease risks,^42^ adjusting for all the covariates would represent over-adjustment and an underestimating of associations. Such an effect was unlikely, however, since the univariate and age-adjusted analyses revealed almost the same positive associations as in the final results.

Contrary to the men whose job characteristics were found to be associated, though weakly, with poor lipid profiles, no positive associations were found for the women. Attitudes toward work and/or perception/expression of job characteristics may be different between genders. The proportion of female employees from these rural settings who sought to develop a career in their occupational activities was not ascertained. Another explanation is that there may be physiological differences related to sex in one’s lipid response to a stressful situation.^43^

We could not take into account the part-time employment, too. It was plausible that more women than men were employed as part time, since a quarter of the women and 11% of the men reported (the questionnaire on the daily activity) to work totally less than 8 hours per day, and this uneven distribution caused, at least in part, gender difference of the findings. We do not know any reports showing the psychometric properties of the demand-control questionnaire in part-time workers. Based on our proxy categories by self-reported working hours, inferred reliability of the control scale was slightly lower in those working less than 8 hours per day than in the counterparts (Chronbach’s alpha = 0.63 for the former, and 0.65 for the latter, respectively). The coefficient levels of psychological demands were not different between the groups (0.67 for both). Since the demand-control questionnaire tries to capture objective work environment,^41^ it is unlikely that the measures produce considerable deviation from the constructs. However, we cannot deny that social roles outside of work interact with job characteristics differently for the employment status.

Although job strain or other forms of chronic work stress seem to have little impact on lipid profiles,^6^ more precise study designs should be adopted before definite conclusions can be drawn for this study question. Prospective studies using lipid levels as outcomes are necessary. Most epidemiologic studies investigating the association (such as ours) were based on a cross-sectional design. With regard to this study question, prospective evidence exists showing that workplace demands are a predictor for the progression of carotid atherosclerosis.^39^ It was also plausible that the stronger effect observed in older men was due to a cumulative effect of adverse job characteristics on athelogenic lipid, because changing job in rural communities was not considered so often as in urban areas. Such an effect can be scrutinized only by repeated measures both of job characteristics and lipid profiles. Interventional studies would also provide a clearer picture. That work stress intervention (i.e., increasing job control) may have a lipid lower effect has been demonstrated.^44^

In conclusion, this cross-sectional analysis indicates a possible association between a psychologically demanding job and an unfavorable lipid profile in Japanese male workers. However, at least in the rural setting, the logical basis for the application of the demand-control model to atherogenic lipids is weak.

## APPENDIX

The Jichi Medical School Cohort Study Group: Akizumi Tsutsumi (Okayama University School of Medicine and Dentistry, Okayama), Atsushi Hashimoto (Aichi Prefectural Aichi Hospital, Aichi), Eiji Kajii (Department of Community and Family Medicine, Jichi Medical School, Tochigi), Hideki Miyamoto (former Department of Community and Family Medicine, Jichi Medical School, Tochigi), Hidetaka Akiyoshi (Department of Pediatrics, Fukuoka University School of Medicine), Hiroshi Yanagawa (Saitama Prefectural University, Saitama), Hitoshi Matsuo (Gifu Prefectural Gifu Hospital, Gifu), Jun Hiraoka (Tako Central Hospital, Chiba), Kaname Tsutsumi (Kyushu International University, Fukuoka), Kazunori Kayaba (Department of Community and Family Medicine, Jichi Medical School, Tochigi), Kazuomi Kario (Department of Cardiology, Jichi Medical School, Tochigi), Kazuyuki Shimada (Department of Cardiology, Jichi Medical School, Tochigi), Kenichiro Sakai (Akaike Town Hospital, Fukuoka), Kishio Turuda (Takasu National Health Insurance Clinic, Gifu), Machi Sawada (Agawa Osaki National Health Insurance Clinic, Kochi), Makoto Furuse (Department of Radiology, Jichi Medical School, Tochigi), Manabu Yoshimura (Kuze Clinic, Gifu), Masahiko Hosoe (Gero Hot-Spring Hospital, Gifu), Masahiro Igarashi, Masafumi Mizooka (Kamagari National Health Insurance Clinic, Hiroshima), Naoki Nago (Tsukude Health Insurance Clinic, Aichi), Nobuya Kodama (Sakugi Clinic, Hiroshima), Noriko Hayashida (Tako Central Hospital, Chiba), Rika Yamaoka (Awaji-Hokudan Public Clinic, Hyogo), Seishi Yamada (Wara National Health Insurance Hospital, Gifu), Shinichi Muramatsu (Department Neurology, Jichi Medical School, Tochigi), Shinya Hayasaka, Shizukiyo Ishikawa (Department of Community and Family Medicine, Jichi Medical School, Tochigi), Shuzo Takuma (Akaike Town Hospital, Fukuoka), Tadao Gotoh (Wara National Health Insurance Hospital, Gifu), Takafumi Natsume (Oyama Municipal Hospital, Tochigi), Takashi Yamada (Kuze Clinic, Gifu), Takeshi Miyamoto (former Okawa Komatsu National Health Insurance Clinic, Kochi), Tomohiro Deguchi (Akaike Town Hospital, Fukuoka), Tomohiro Saegusa (Sakuma National Health Insurance Hospital, Shizuoka), Yoshihiro Shibano (Saiseikai Iwaizumi Hospital, Iwate) Yoshihisa Ito (Department of Laboratory Medicine, Asahikawa Medical College, Hokkaido), and Yosikazu Nakamura (Department of Public Health, Jichi Medical School, Tochigi).
